# Investigation of population-based mental health staffing and efficiency-based mental health productivity using an information-theoretic approach

**DOI:** 10.1371/journal.pone.0256268

**Published:** 2021-08-16

**Authors:** Matt Boden, Clifford A. Smith, Jodie A. Trafton

**Affiliations:** 1 Program Evaluation and Resource Center, VHA Office of Mental Health and Suicide Prevention, Menlo Park, California, United States of America; 2 Center for Innovation to Implementation, VHA Palo Alto Health Care System, Menlo Park, California, United States of America; 3 VHA Office of Mental Health and Suicide Prevention, Washington, D.C., United States of America; 4 Department of Psychiatry and Behavioral Sciences, Stanford University Medical School, Stanford, California, United States of America; University of California San Diego School of Medicine, UNITED STATES

## Abstract

**Background:**

Healthcare systems monitor and improve mental health treatment quality, access, continuity and satisfaction through use of population-based and efficiency-based staffing models, the former focused on staffing ratios and the latter, staff productivity. Preliminary evidence suggests that both high staffing ratios and moderate-to-high staff productivity are important for ensuring a full continuum of mental health services to indicated populations.

**Methods & findings:**

With an information-theoretic approach, we conducted a longitudinal investigation of mental health staffing, productivity and treatment at the largest integrated healthcare system in American, the Veterans Health Administration (VHA). VHA facilities (*N* = 140) served as the unit of measure, with mental health treatment quality, access, continuity and satisfaction predicted by facility staffing and productivity in longitudinal mixed models. An information-theoretic approach: (a) entails the development of a comprehensive set of plausible models that are fit, ranked and weighted to quantitatively assess the relative support for each, and (b) accounts for model uncertainty while identifying best-fit model(s) that include important and exclude unimportant explanatory variables. In best-fit models, higher staffing was the strongest and most consistent predictor of better treatment quality, access, continuity and satisfaction. Higher staff productivity was often, but not always associated with better treatment quality, access, continuity and satisfaction. Results were further nuanced by differential prediction of treatment by between- and within-facility predictor effects and variable interactions.

**Conclusions:**

A population-based mental health staffing ratio and an efficiency-based productivity value are important longitudinal predictors of mental health treatment quality, access, continuity and satisfaction. Our longitudinal design and use of mixed regression models and an information-theoretic approach addresses multiple limitations of prior studies and strengthen our results. Results are discussed in terms of the provision of mental health treatment by healthcare systems, and analytical modeling of treatment quality, access, continuity and satisfaction.

## Introduction

Multiple interacting factors impact the quality, access to and satisfaction with mental health treatment in healthcare systems. As mental health staff are the direct providers of treatment, the number of staff available and the amount and type of treatment they provide are important proximal factors. Population-based staffing models identify the number of staff needed to provide care to the population of eligible service-users [1,2]. Thus, a population-based staffing model is derived from the staffing ratio (e.g., full-time-equivalent (FTE)-per-1000 treated outpatients), which can be tracked and changed to improve the quality of mental health treatment. Alternatively, an efficiency-based staffing model [3] focuses on the amount and type of work required by staff to provide care to service-users. Thus, productivity of staff is a central focus of these models.

Reliance upon efficiency-based models, and specifically, modification of productivity, to improve treatment outcomes is appealing given the high direct costs of adding and maintaining staff. Yet, we have found that a population-based model is of greater importance than an efficiency-based model for ensuring treatment quality and population access to the full continuum of mental health services [4]. Specifically, using data from the from the Veterans Health Administration (VHA), the largest integrated mental health treatment program in the United States, we found that mental health staffing ratios (a) moderately (and positively) predicted mental health treatment quality, access, continuity and satisfaction, and (b) were stronger predictors of these outcomes than were staff productivity and wait-times. Productivity predicted treatment outcomes to a moderate degree when accounting for staffing ratio in a subset of analyses. These results provide evidence that productivity, and especially staffing ratios, are important predictors of mental health treatment. Yet, the cross-sectional study design and the traditional frequentist hypothesis testing approach utilized limited the strength of this evidence.

In the current study, we advance the empirical literature on mental health staffing, staff productivity and treatment while addressing the limitations of Boden and colleagues [4] and other studies on mental health staffing models (e.g., which focus on the psychiatry workforce [3] or on community mental health caseload calculations [5]) by conducting a longitudinal investigation of VHA mental health staffing, staff productivity and treatment based on an information-theoretic approach. Longitudinal analyses that include repeated measurements of individual respondents, which in this study are VHA facilities: (a) reduce the likelihood that results are not biased by anomalous results obtained at one moment in time (i.e., cross-sectional design), and (b) allow for examination of both within- and between-facility variation [6]. Regarding the latter, variation in staffing and productivity within a given facility over time and between facilities might each be associated with variation in mental health quality, access and satisfaction, but to different degrees, and perhaps, in different directions. Generalized linear mixed models (GLMM), which allow for the inclusion of both fixed and random predictor effects, can examine these different sources of variation in relation to a given outcome [7,8].

An information-theoretic approach entails the development of a comprehensive set of plausible models that are fit, ranked and weighted (based on the calculation of a specified fit statistic such as Akaike information criterion [AIC]) to quantitatively assess the relative support for each model [9,10]. Unlike a traditional frequentist approach (e.g., implemented through null-hypothesis testing), an information-theoretic approach accounts for model uncertainty while identifying a best-fit model or models that include important and exclude unimportant explanatory variables [10,11]. Through model averaging of models that fit the data reasonably well, robust parameter estimates and a measure of the importance of individual predictors are obtained. Thus, this approach is useful here as there are numerous combinations of fixed and random between- and within-facility predictor effects that might be included in our longitudinal models, the importance of which is unknown a-priori. Furthermore, an information-theoretic approach does not suffer from many of the noted limitations and problems associated with a traditional frequentist approach [12]. Importantly, testing of a-priori models that do not actually fit the data well can provide misleading results [9]. In cases in which models are modified to improve fit, researchers often change models in ways that are idiosyncratic or less than empirically sound (e.g., step-wise regression). This is especially problematic when many parameters might be included in a given model, such is the case with our analysis. Rather, we use an empirically rigorous approach to fit and test models.

Our study centers on two research questions. First, is mental health staffing and staff productivity associated with mental health treatment quality, access, continuity and satisfaction over time? This question is answered by determining whether best-fit models include staffing ratio and/or staff productivity (and their interaction) as substantive predictors of mental health treatment quality, access, continuity and satisfaction (i.e., determined by effect size and range of confidence intervals for parameter estimates). Mental health treatment quality, access, continuity and satisfaction are operationalized by VHA’s core mental health access/quality metrics: Strategic Analytics for Improvement and Learning (SAIL) metrics [13]. Second, to what extent are staffing and staff productivity incrementally important predictors of mental health SAIL metrics? To answer this question, we compare effect size and range of confidence intervals for parameter estimates of staffing ratio and staff productivity when obtained from model averaging of best-fit models. We address our research questions in terms of total mental health staffing ratio and staff productivity, including all types of providers of direct clinical care (e.g., psychiatrists, psychologists, social workers, counselors, clinical nurse specialists).

## Materials and methods

VHA healthcare facilities (*N* = 140) served as the unit of measure. All variables calculated/collected at the level of VHA facility are updated quarterly as part of program evaluation and improvement. Analyses reported below included data obtained for quarters 1–4 of fiscal years 2016 through 2018 (approximately October 2015 through September 2018) for a total of 12 time-points.

The Stanford IRB determined this study to be non-research, as it was a quality improvement effort to directly guide operational decision making about resource allocation that would optimize clinical care for VHA patients. Identifiable medical record data was accessed initially for operational planning to ensure health care access and quality for VHA patients as authorized by HIPAA. An aggregate, de-identified database was utilized for all analyses.

### Measures

#### Staffing ratio

Outpatient mental health staffing ratios provided a direct estimate of the total FTE providing direct clinical care in outpatient mental health settings per 1000 treated mental health outpatients. Quarterly staffing ratio values were calculated by dividing mental health outpatient FTE by mental health outpatients then multiplying by 1000. The numerator, mental health outpatient FTE included the proportion of FTE of all staff who provided direct clinical care to patients in specialty mental health clinics, including but not limited to primary care mental health integration (PCMHI), substance use disorder, posttraumatic stress disorder, and mental health intensive case management clinics. All provider types were included (psychiatrists, psychologists, social workers, clinical nurse specialists, nurse practitioners, physician assistants, clinical pharmacists, other medical doctors, marriage and family therapists, licensed professional counselors, other counselors, registered nurses, pharmacists, other staff).

FTE for individual staff were calculated by pay period by multiplying (a) the proportion of patient encounters that occurred in outpatient mental health clinics, by (b) hours worked in a pay period, by (c) percentage of the staff member’s time allocated to direct clinical care, divided by (d) 80 possible hours in a pay period. We summed individual staff FTE by facility, divided by the denominator, mental health outpatients in the prior four quarters, and multiplied by 1000 to obtain staffing ratios by pay period. We averaged staffing ratios by pay period to obtain quarterly values.

#### Productivity

Outpatient mental health staff productivity was calculated to reflect productivity of clinicians (all provider types) as they provide care to outpatients in mental health clinics, rather than productivity across all assigned clinical duties. Work relative value units (wRVUs from Centers for Medicare and Medicaid Services and VHA-imputed values) were identified for all providers with outpatient mental health FTE from patient encounters in outpatient mental health clinics (see ‘a’ above). All outpatient mental health wRVUs for all providers within a facility were summed across pay periods within a given quarter. This total wRVUs value was divided by the sum of outpatient mental health clinical hours worked by providers (from above, a*b*c) and multiplied by 466.75 (bookable hours in the quarter).

#### Mental health SAIL composites

Mental health SAIL variables include Population Coverage, Continuity of Care and Experience of Care mental health SAIL composites, and the Mental Health Domain score, a meta-composite of the three composite measures [13]. The Population Coverage composite is comprised of component metrics that assess access to care, including the proportions of veterans with identified mental disorders who receive specialty mental health services promised by the VA uniform mental health service package (18). The Continuity of Care composite is comprised of process quality of care metrics assessing whether patients initiate and engage in evidence-based treatments at a particular frequency, and the extent to which mental health services are provided in a coordinated, proactive manner. The Experience of Care composite includes component metrics that are veteran and provider survey responses to questions assessing access, quality, and coordination of care. Individual metrics that comprise SAIL composites are sometimes updated at the start of a SAIL measurement year to address emerging care delivery policy and national guidance across the VHA mental healthcare system.

To capture stable program characteristics, composite measures include one new quarter of data and he three quarters of prior data [13]. To facilitate both within- and between-facility comparisons, scores are standardized across facilities each four-quarter SAIL measurement-year using the distribution of facility scores in the baseline quarter (last quarter of a fiscal year). Positive scores indicate better than average performance and negative scores worse than average performance relative to other facilities and, within facilities, relative to performance in the baseline quarter. In a given quarter, a facility score of one would represent a one standard deviation above the facility average, and in practical terms signify that the scores on a combination of component metrics locate that facility at approximately the 84^th^ percentile of all facilities. Similarly, a score of negative one, or one standard deviation below the facility average would locate the facility in the approximately the 16^th^ percentile of all facilities based on a combination of component metrics.

#### Facility complexity

From the VHA facility complexity model [14], facility complexity was rated on a five-level scale based on factors such as provision of complex clinical programs, intensive care, operative complexity, and allocation of research funds.

### Data analysis

No data was missing from any variable at any time-point. Preliminary to our main analyses, we calculated descriptive statistics, between-subject correlations by correlating the average (across time-points) value of each variable, and within-subject correlations based on residual variance from multi-level models [15].

In our primary analyses, we examined longitudinal associations between SAIL mental health metrics, and staffing and productivity using linear mixed regression models and an information-theoretic approach. In each of our four sets of analyses, the response variable was a mental health SAIL metric, and the predictors were various combinations of fixed and random effects of time, staffing ratio, productivity, their interactions, and facility complexity. Models with (a) three-way interactions, (b) more than two random slopes, or (c) a random slope for any interaction effect tended not to converge, and thus, were not included in any model set. Staffing ratio and productivity were decomposed into between- and within-facility effects in some models, and in those models, both between- and within-facility effects were included. In no models did we include both an overall effect and decomposed between- and within-facility effects.

A set (i.e., predicting a single SAIL mental health metric) included approximately 850 models, which were weighted, compared, and ranked based on AIC corrected for small sample size (AICc; [16]). Using an established criterion to identify models with substantial support (ΔAIC_C_<4; [9]), we identified and report a subset of top models out of each set, and parameter estimates, standard errors and confidence intervals obtained through natural averaging across the subset of top models. To evaluate goodness-of-fit of top models, we calculated pseudo-*R*^*2*^ as proposed by Snijders and Bosker [17]. The relative importance and strength of each predictor was determined by (a) the likelihood the predictor was included in the subset of top models (assessed by summing Akaike weights of models that include the predictor out of subset of top models), and (b) parameter estimate size and 95% confidence intervals (CI).

All predictors were centered and standardized, thus allowing us to directly compare parameter estimates after model averaging and interpret main effects in the presence of interactions, for which predicted scores were plotted and interpreted [10]. All analyses were conducted in R using the lme4 [7], MuMin [18], ggplot [19], effects [20], and sjstats [21] packages.

## Results & discussion

### Results

Descriptive statistics and zero-order correlations between variables are shown in Table 1.

**Table 1:** Between-facility correlations (above the diagonal) and within-facility correlations (below the diagonal) and descriptive statistics for all variables included in the study.

	1	2	3	4	5	6
1) Staffing		-.32	.59	.47	.43	.44
2) Productivity	.30		.01	.21	-.13	-.08
3) SAIL Mental Health Domain	.15	.07		.68	.78	.81
4) SAIL Population coverage	-.04	.08	.41		.22	.26
5) SAIL Continuity of care	.13	.05	.75	-.03		.61
6) SAIL Experience of care	.11	-.03	.61	.16	-.02	
*Mean*	7.34	449.12	.16	.05	.24	.06
*Min*	4.66	313.84	-2.15	-2.17	-1.86	-2.09
*Max*	14.71	659.65	2.84	2.70	2.64	3.13
*SD within*	1.54	73.91	.99	.99	1.04	.98
*SD between*	1.48	65.91	.90	.95	.82	.86

Across facilities collapsed over time-points (between-facility correlations): (a) higher staffing ratio was associated with lower productivity and with higher SAIL mental health scores to a moderate degree (mean correlation coefficient [MCC] = .48); and (b) higher productivity was generally associated with both higher and lower mental health SAIL scores to a small degree (MCC = .00). Within a given facility over time (within-facility correlations), (a) higher staffing ratio was associated with higher productivity to a small-to-moderate degree (MCC = .30), and mental health SAIL scores to a small degree (MCC = .09); and (b) higher productivity was associated with higher SAIL mental health scores to a small degree (MCC = .04).

In our primary analyses, two-to-three models met top-model criteria (ΔAIC_C_<4) for each mental health SAIL response variable (see Table 2).

**Table 2:** Top models (ΔAIC_C_<4) predicting SAIL mental health metrics.

	Predicting SAIL Mental Health Domain					
Model	df	AIC_C_	ΔAIC_C_	Weight	Rank	*R* ^*2*^
1p) T* + SW + SB + P*	12	1890.17	.00	.60	1	.90
2p) T* + SW + SB + P* + SB:P	13	1892.85	2.68	.16	2	.90
3p) T* + SW + SB + P* + T:SW	13	1893.29	3.12	.13	3	.90
	Predicting SAIL Population Coverage					
Model	df	AIC_C_	ΔAIC_C_	Weight	Rank	*R* ^*2*^
4p) T* + SW* + SB + PW + PB	13	463.62	.00	.67	1	.96
5p) T* + SW* + SB + PW + PB + T:PW	14	467.48	3.86	.10	2	.96
6p) T* + SW* + SB + PW + PB + T:SW	14	467.49	3.87	.10	3	.96
	Predicting SAIL Continuity of Care					
Model	df	AIC_C_	ΔAIC_C_	Weight	Rank	*R* ^*2*^
7p) T* + S + P* + S:P	12	3529.58	.00	.41	1	.74
8p) T* + S + P*	11	3529.71	0.13	.39	2	.73
	Predicting SAIL Experience of Care					
Model	df	AIC_C_	ΔAIC_C_	Weight	Rank	*R* ^*2*^
9p) T* + SW* + SB + P	12	2473.44	.00	.42	1	.85
10p) T* + SW* + SB + P + T:SW	13	2474.00	0.55	.32	2	.85
11p) T* + SW* + SB + P + T:P	13	2476.26	2.82	.10	3	.85

Notes:

*Denotes that a random slope was included for the predictor.

All models included a random intercept. T = Time. S = Staffing (overall). SB = Staffing (between facilities). SW = Staffing (within facilities). P = Productivity (overall). PB = Productivity (between facilities). PW = Productivity (within facilities). *R*^*2*^ = pseudo-*R*^*2*^ as proposed by Snijders and Bosker [17].

The top models predicting Mental Health Domain (model 1p) and Population Coverage (model 4p) had a greater than 60% probability of being the top model, whereas the top model predicting Continuity of Care and Experience of Care had a greater than 40% probability. Yet, model selection uncertainty for the latter two models was balanced by the top models together having an 80 to 84% probability of being the top model (e.g., ∑ Akaike weight for models predicting Experience of Care = .84). The variance explained by the two-to-three top models ranged from a low of .73 (predicting Continuity of Care) to a high of .96 (predicting Population Coverage).

Top models included random intercepts, and random slopes for time (see Table 2). Other than for top models predicting Continuity of Care, all top models included staffing ratios decomposed into between- and within-facility effects. Top models for Population Coverage and Experience of Care, but not Mental Health Domain, included a random slope for within-facility staffing ratio. An overall staffing ratio (i.e., not decomposed into between- and within-facility effects) was included in top models for Continuity of Care. Other than for Population Coverage, top models for SAIL mental health composites included an overall effect for productivity, with an associated random slope in models for Mental Health Domain and Continuity of Care. Top models for Population Coverage included productivity decomposed into between- and within-facility effects. Seven out of eleven top models included interactions, five of which were interactions with time. No top models included facility complexity as a predictor.

Time, staffing and productivity all were relatively important predictors of mental health SAIL metrics, as they appear in all top models (see Table 3).

**Table 3:** Predictor sum of weights (ordered by), parameter estimates, SE and 95% confidence interval (CI) after full model averaging of the top models (ΔAIC_C_<4), separately assessing each SAIL mental health metric.

	Predicting SAIL Mental Health Domain					
Variable(s)	Weight	Estimate	SE	95% CI		
I		.17	.06	.05	,	.28
T	.88	.00	.02	-.04	,	.04
Sb	.88	.57	.06	.45	,	.68
Sw	.88	.09	.01	.06	,	.11
P	.88	.21	.04	.14	,	.28
Sb:P	.16	-.01	.03	-.14	,	-.004
T:Sw	.13	.00	.01	.01	,	.04
	Predicting SAIL Population Coverage					
Variable(s)	Weight	Estimate	SE	95% CI		
I		.05	.06	-.08	,	.18
T	.86	-.09	.02	-.13	,	-.05
Sw	.86	.02	.01	-.01	,	.04
Sb	.86	.56	.07	.43	,	.69
Pw	.86	.02	.01	.01	,	.04
Pb	.86	.38	.07	.25	,	.51
T:Pw	.10	.00	.01	-.03	,	-.003
T:Sw	.10	.00	.01	.004	,	.03
	Predicting SAIL Continuity of Care					
Variable(s)	Weight	Estimate	SE	95% CI		
I		.23	.06	.11	,	.36
T	.80	.15	.03	.09	,	.20
S	.80	.39	.05	.29	,	.49
P	.80	.18	.05	.08	,	.28
S:P	.41	-.05	.06	-.18	,	-.03
	Predicting SAIL Experience of Care					
Variable(s)	Weight	Estimate	SE	95% CI		
I		.06	.07	-.07	,	.19
T	.84	-.06	.02	-.10	,	-.01
Sw	.84	.03	.02	-.01	,	.07
Sb	.84	.41	.07	.28	,	.53
P	.84	.06	.03	.01	,	.11
T:Sw	.32	.01	.02	.01	,	.06
T:P	.10	-.01	.02	-.08	,	-.01

Notes: T = Time. S = Staffing (overall). Sb = Staffing (between facilities). Sw = Staffing (within facilities). P = Productivity (overall). Pb = Productivity (between facilities). Pw = Productivity (within facilities).

Based on parameter estimates, the strongest predictor of Mental Health Domain, Population Coverage and Experience of Care was between-facility staffing. Staffing was also the strongest predictor of Continuity of Care, but as an overall score. Facilities with higher staffing had higher metric scores. Within-facility staffing ratios tended to be less strongly associated with mental health SAIL metrics, and 95% confidence intervals for these effects included zero other than for Mental Health Domain. Within-facility increases in staffing over time tended to be associated with increases in mental health SAIL metrics. Productivity was the next strongest predictor of Mental Health Domain (overall score), Population Coverage (between-facility score), and Continuity of Care (overall score), and predicted Experience of Care (overall score) to the same extent as time. Similar to staffing, higher levels of productivity were associated with higher mental health SAIL metric scores.

**Fig 1:**
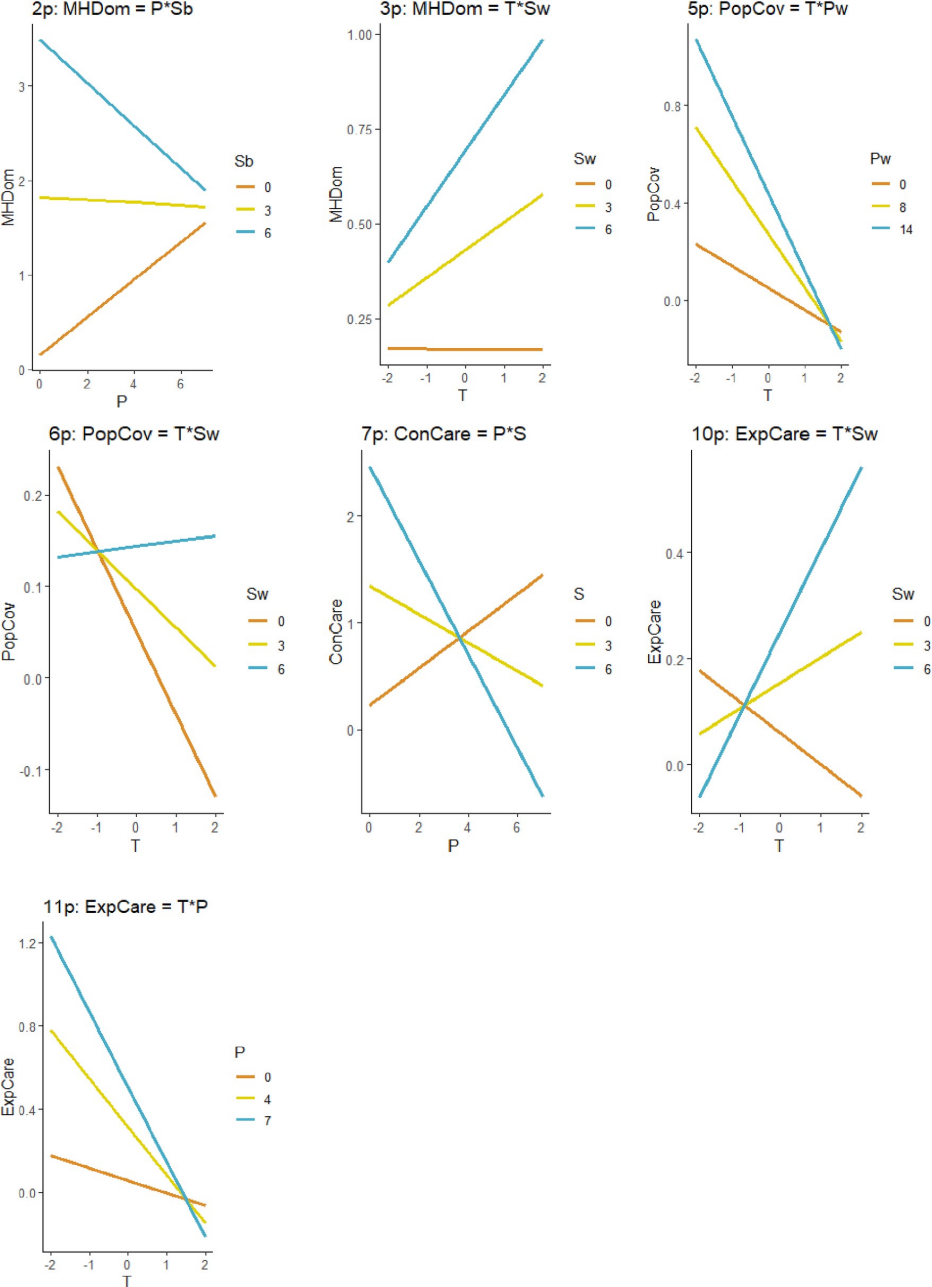
Interactions between predictors in top models. T = Time. S = Staffing (overall). Sb = Staffing (between facilities). Sw = Staffing (within facilities). P = Productivity (overall). Pb = Productivity (between facilities). Pw = Productivity (within facilities).

Interaction effects were relatively less important than the effects of individual predictors, and average parameter estimates were small in size. Predicting Mental Health Domain: (a) facilities high in staffing and low in productivity had very high scores that decreased with increasing productivity, whereas facilities low in staffing had moderate scores that increased with increasing productivity (Fig 1, Model 2p); and (b) within a given facility, higher than average staffing was associated with moderate scores that increased over time, whereas lower than average staffing was associated with moderate and stable scores (Model 3p).

Predicting Population Coverage: (a) within a given facility, higher than average productivity was associated with high scores that decreased over time, whereas lower than average staffing was associated with moderate scores that decreased over time (Model 5p); and (b) within a given facility, higher than average staffing was associated with stable and moderate scores, whereas lower than average staffing was associated with moderate scores that sharply decreased over time (Model 6p). Predicting Continuity of Care, facilities low in overall staffing tended to have moderate scores that increased with increasing productivity, whereas facilities high in overall staffing tended to have high scores that sharply decreased with increasing productivity (Model 7p). Predicting Experience of Care Coverage: (a) within a given facility, higher than average staffing was associated with moderate scores that sharply increased over time, whereas lower than average staffing was associated with moderate scores that decreased over time (Model 10p); and (b) facilities with high overall productivity had high scores that sharply decreased over time, whereas facilities with low overall productivity tended to have moderate and relatively stable scores over time (Model 11p).

### Discussion

Using an information-theoretic approach, we obtained evidence that a population-based mental health staffing ratio and an efficiency-based productivity value are important longitudinal predictors of mental health treatment quality, access and satisfaction. This study is among the first to demonstrate associations between population-based staffing levels and mental health treatment quality, access, continuity and satisfaction [4], and the first we know to demonstrate these longitudinal associations. Our longitudinal design helped us to avoid bias associated with potentially anomalous results at any one time point. Furthermore, using longitudinal mixed models, we were able to account for correlations between values at different time-points and to separate between- and within-facility predictor effects, which tended to differ in size and importance. The use of longitudinal mixed models paired with an information-theoretic approach increases our confidence that our results are reliable and valid. The information-theoretic approach, which avoids many of the problems of a frequentist approach [12], provided a systematic and valid means by which to identify best-fit models and important explanatory variables. This was important given the numerous combinations of between- and within-predictor effects and random and fixed effects that might otherwise have been (inaccurately) specified a-priori. The results of the study provide additional strong evidence that a population-based mental health staffing ratios and efficiency-based adjusted productivity are important correlates of mental health treatment.

In top models, mental health staffing ratios, and especially between-facility ratios, were the strongest predictor of all mental health SAIL metrics. As expected, facilities with higher versus lower staffing tended to have higher metric scores. Furthermore, above average staffing in a given facility over time tended to be associated with higher levels of mental health SAIL metrics, though, this effect was smaller than the between-facility effect. Interactions between within-facility staffing and time demonstrated increasing quality of (i.e., Mental Health Domain) and satisfaction with (i.e., Experience of Care) mental health treatment across time when a given facility had above average staffing and decreasing quality and satisfaction when a facility had below average staffing. Similarly, access to treatment (i.e., Population Coverage) remained stable over time when a given facility had above average staffing but decreased when a facility had average or below average staffing.

It is no surprise that mental health staffing was a consistent and strong predictor of mental health treatment quality, access, continuity and satisfaction. Having more mental health providers of direct clinical care allows more patients needing specialized mental health care to be treated, and in a timely and continuous manner using the most effective treatments available. Following, patients (and providers) are satisfied with the resulting high-quality care. Yet, our results further suggest that productivity and, in some cases, the interaction between staffing and productivity play roles in mental health treatment quality, access, continuity and satisfaction. Higher levels of productivity were associated with higher mental health SAIL metric scores, but mostly as an overall effect. In other word, between-facilities and within-facilities across time (together as a single effect), higher levels of productivity predicted higher overall quality, care continuity and satisfaction. Interestingly, as demonstrated by interactions with time, above average productivity within a given facility over time was associated with decreasing access (i.e., Population Coverage), and high overall productivity over time was associated with decreasing satisfaction (i.e., Experience of Care). In both cases, lower productivity within a given facility or overall, was associated with decreases in mental health SAIL scores, but to a lesser extent than above average/high overall productivity.

When adjusting for variance shared between them, both population-based mental health staffing and efficiency-based adjusted productivity consistently predicted mental health treatment quality, access, continuity and satisfaction. Yet, these conclusions are further nuanced by interactions between staffing and productivity. Facilities with high staffing levels had high Mental Health Domain scores despite having low productivity. Alternatively, low productivity in low staffed facilities was equated with lower Mental Health Domains scores. Increasing productivity helped low staffed facilities to achieve higher and moderate Mental Health Domain scores, on par with facilities with high and average levels of staffing. Thus, greater efficiency appears to counteract the negative impact of low staffing on mental health treatment quality (though, high levels of staffing always predicted higher mental health treatment quality). A somewhat similar pattern was true of Continuity of Care scores when examining overall staffing and productivity: among facilities with low staffing, high overall productivity predicted above average scores, whereas low overall productivity predicted moderate scores. Interestingly, highly staffed facilities had lower scores when they also had high versus low overall productivity. Thus, greater efficiency (more procedures completed per time worked) appears to be associated with mental health treatment of higher quality and greater continuity when staffing is low versus high. In facilities with low staffing, greater efficiency may be associated with focused care tailored to the individualized needs of patients. For example, patients may receive specialized services for their mental conditions (e.g., cognitive processing therapy for posttraumatic stress disorders) from a small set of core providers who regularly communicate. Alternatively, increased efficiency in highly staffed facilities may be manifested as a higher volume of services that are less continuous, with less communication and contact between the large number of providers involved in any one case.

Using an innovative approach, our study gained additional evidence supporting a relation between mental health treatment quality, access, continuity and satisfaction and population-based staffing and efficiency-based productivity [4]. Replication of these results using data from other timeframes, for example, will strengthen our confidence that the best fit models found here represent the data more broadly. Furthermore, unequivocal evidence regarding the role of staffing and productivity on mental health treatment will come from studies that explicitly manipulate staffing and productivity (or, through natural experiments, isolate changes in staffing and productivity) and examine resulting changes in mental health treatment quality, access, continuity and satisfaction. The correlational nature of ours study limits us from drawing conclusions regarding causal relations between variables.

As we conducted our study within VHA, the largest integrated healthcare system in the United States, our results may be most generalizable to other large healthcare systems in the United States. Furthermore, unique characteristics of VHA’s structure, service provision, and patient population may also narrow the generalizability of results. Thus, our results should be applied with caution to understand staffing and productivity of mental health providers as it relates to mental health treatment outside of VHA until research is conducted in other healthcare systems. Such research may benefit from examining relations between staffing/productivity and mental health performance as assessed by measures outside of VHA, such as Health Plan Employer Data and Information Set (HEDIS), Centers for Medicare and Medicaid Services (CMS), and National Committee for Quality Assurance measures [22]. Indeed, mental health SAIL composite metrics include four HEDIS component metrics, and multiple studies have utilized HEDIS measures with VHA mental health populations [23,24]. We note that current program evaluation and research efforts by the VHA Office of Mental Health and Suicide Prevention are focused on surmounting challenges (e.g., differences in electronic medical record coding practice within and outside of VHA) to integrate additional metrics into VHA mental healthcare program evaluation.

We limited our study to examining staffing and productivity as it pertains to all provider types considered together. Yet, in an integrated healthcare system where mental health treatment is team-based and a diverse range of treatments are needed to address the mental health needs of patients, mental health staff of various types serve unique (e.g., prescribers are experts in biomedical treatments, therapists focus on the provision of psychosocial treatments) and overlapping roles (e.g., therapists and prescribers may engage in some form of case management). Thus, it will be useful to evaluate whether multiple staff types are associated with high quality mental health care, as found in cross-sectional analyses [4]. It may also prove useful to investigate the impact of administrative staffing on mental health treatment outcomes. Many providers engage in duties other than direct clinical care, such as teaching, research and administration. Staffing ratios and productivity variables included in our study exclude provider time dedicated to duties other than direct clinical care. VHA facilities may vary in how they characterize administrative duties, in provider time allocated to administrative duties, and in the number of ancillary administrative staff in mental health clinics. Future research will benefit from characterizing and investigating the impact of gaps in administrative staffing on the productivity of staff engaged in clinical duties.

Composite measures such as MH SAIL metrics are useful for summarizing large amounts of data to provide concise yet comprehensive overviews of healthcare performance. These measures provide clear and accessible information on a medical facility’s performance relative to other facilities and across time, which can be leveraged by stakeholder at different levels of the organization for tracking, planning, decision making, and intervention/improvement efforts [13]. Yet, future research will also benefit from examining relations between staffing and productivity and mental health SAIL component measures [25], which can inform continuous quality improvement efforts at the facility level. Mapping variability in relations between staffing and productivity and SAIL component metrics may prove useful for identifying specific areas where initiatives intended to increase staffing and optimize productivity may be leveraged to improve performance. It will also be useful to move beyond mental health SAIL metrics to examine downstream clinical outcomes. At least one study has examined associations between staffing and suicide rates in the VHA [26], though, this study was cross-sectional and did not also examine the role of productivity. Longitudinal investigations, potentially utilizing and information-theoretic approach will provide needed and complimentary data regarding whether the benefits of staffing and productivity on mental healthcare performance extends to consequential downstream outcomes.

## Conclusions

Our results have clear implications for mental health treatment. Additional staffing will be associated with higher quality treatment, and greater access to continuity of and satisfaction with treatment. Higher efficiency of staffing though, appears to be especially beneficial for facilities staffed at lower levels, and facilities with efficiency that is too high (i.e., too many procedures performed per time spent with patient) may be at risk of providing subpar mental health treatment over time. These conclusions are consistent with anecdotal reports that too high of productivity is associated with chaotic work environments and provider burnout. Based on these and prior results [4], we hypothesize that mental health programs should focus on building capacity via increased staffing then work to optimize productivity. Due to time and effort directed to training and integrating new staff, productivity might decrease initially, but ultimately return to optimized levels (not low or excessively high) as workload is balanced amongst staff who provide care to new and existing patients. Additional research is needed to directly test these hypotheses.

Our findings also have implications for the modeling of mental health treatment quality, access, continuity and satisfaction. The commonly used, frequentist hypothesis testing approach for which models are specified a-priori, may be less than ideal when multiple, potentially interacting predictors are thought to be associated with mental health treatment quality, access, continuity and satisfaction. This appears to be especially true of longitudinal mixed models, which can include fixed and random effect, and between- and within-variable effects. Indeed, though we included only three predictors in our models in addition to time, the best fitting models of mental health treatment quality, access, continuity and satisfaction were not what we had expected, and likely not among the models we would have considered for testing. The information-theoretic approach and, potentially, other non-frequentist approaches [27] are ideally suited to model testing in these circumstances. We hope that information-theoretic, which has gained traction in other fields, is more readily applied to the area of mental health.
